# Chemoprevention utility of silibinin and Cdk4 pathway inhibition in *Apc*^*−/+*^ mice

**DOI:** 10.1186/1471-2407-13-157

**Published:** 2013-03-27

**Authors:** Baktiar O Karim, Ki-Jong Rhee, Guosheng Liu, Dongfeng Zheng, David L Huso

**Affiliations:** 1 Department of Molecular and Comparative Pathobiology, The Johns Hopkins University, 733 N. Broadway St, BRB Bldg #849, Baltimore, MD 21205, USA; 2 Department of Biomedical Laboratory Science, Yonsei University, 1 Yonseidae-gil, Wonju, Gangwon-do 220-710, Republic of Korea

**Keywords:** Apc, Cdk4 inhibition, Chemoprevention, Silibinin

## Abstract

**Background:**

Colorectal cancer (CRC) is the second leading cause of death from cancer in the United States. Colorectal cancers have a prolonged latency following initiation that may span decades providing ample time for implementing a chemoprevention strategy that could block or reverse the progression to CRC. Cdk4 pathway alterations have been linked to a number of cancers including CRC. In these experiments we focused on the Cdk4 pathway and its role in intestinal tumorigenesis as a possible target in chemoprevention strategies.

**Methods:**

We evaluated the effect of *Cdk4* blockade on the prevention of intestinal tumor formation by crossing *Cdk4*^*−/−*^ mice to *Apc*^*−/+*^ mice. In addition, we tested the effect of the dietary compound silibinin on the Cdk4 pathway in *Apc*^*−/+*^ mice and HT-29 colon cancer cells in culture.

**Results:**

*Cdk4*^*−/−*^ mice backcrossed to *Apc*^*−/+*^ mice reduced intestinal adenoma formation compared to *Apc*^*−/+*^ controls. Silibinin effectively targeted the Cdk4 pathway causing hypophosphorylation of the retinoblastoma protein, inhibited cell growth, and induced apoptosis. As a result silibinin blocked the development of intestinal adenomas by 52% in this genetic model (*Apc*^*−/+*^ mice) of early events in colorectal cancer formation. No toxic abnormalities were detected in mice which received silibinin.

**Conclusions:**

Modification of the Cdk4 pathway using a natural plant-derived compound such as silibinin may be a useful chemopreventive strategy for colorectal carcinomas.

## Background

Disruption of cell cycle regulation through alterations in the Cdk4 pathway appears to play an important role in the development of a variety of cancers including colorectal cancer [1,2]. It is now clear that colorectal cancers begin with intestinal epithelial cell clones that lose the function of the *Apc* pathway (gatekeeper function) [3,4]. *Apc* is part of the cellular Wnt signaling pathway and is an upstream regulator of many components of the Cdk4 pathway [5,6]. Loss of *Apc* function acts to upregulate c-Myc and cyclin D1 expression [7-9]. c-Myc is a direct activator of *Cdk4* as the *Cdk4* gene has 4 conserved c-Myc binding sites in its promoter [10]. Cdk4 is a key regulator of the cell cycle, and when Cdk4 becomes active by binding to cyclin D1, it allows cells to enter the G_1_ phase of the cell cycle and progress towards the S phase by phosphorylating retinoblastoma (Rb) protein. This leads to release of E2F transcription factors that function to activate the transcription of genes involved in DNA synthesis [8,11]. Therefore, *Apc* mutations, acting in part through the activation of the Cdk4 pathway, play a critical role early in intestinal tumorigenesis [12,13].

Many studies suggest the utility of natural compounds as chemopreventive agents against CRC [14,15]. Some compounds derived from natural sources have been shown to inhibit the cell cycle at points regulated by various components of the Cdk4 pathway, blocking proliferation of cancer cells [16-18]. For example, milk thistle, also referred to as St. Mary's thistle, lady thistle, or holy thistle, contains polyphenolic flavanoid antioxidant compounds composed mainly of silibinin. For several decades, silymarin, a compound derived from milk thistle and related to silibinin, has been used empirically around the world for the treatment of liver cirrhosis [19]. Recent studies suggest that silibinin has antiproliferative activity *in vitro* and *in vivo* and causes G_1_ arrest of the cell cycle (regulated by the Cdk4 pathway) [17].

In this study, we examined the Cdk4 pathway and its blockade in the context of *Apc* mutation to determine the potential for targeting this pathway in a chemopreventive strategy for colorectal cancer. In these experiments, we compared the effects of Cdk4 pathway blockade on the number and size of adenomas in the *Apc*^*−/+*^*Cdk*^*+/+*^ with *Apc*^*−/+*^*Cdk4*^*−/−*^ mice to characterize the role of the Cdk4 pathway in tumor development *in vivo*. Furthermore, we found silibinin effectively opposes Cdk4 pathway activation at several points and administration as a chemopreventive agent resulted in reduced cell proliferation, increased apoptosis, and reduced adenoma development in *Apc*^*−/+*^ mice.

## Methods

### Animal model

C57BL/6-*Apc*^*tm1.Δ716*^ mice were generously provided by Drs. Vogelstein and Kinzler [20]. *Apc* heterozygote (*Apc*^*−/+*^) offspring were genotyped at three weeks of age. A *Cdk4*-null mutation, *Cdk4*^*tm1Kiyo*^ (*Cdk4*^*−/−*^) mice were provided by Kiyokawa [21]. To generate double mutant *Apc*^*−/+*^*Cdk4*^*−/−*^ mice, *Apc*^*−/+*^ were crossed with *Cdk4*^*−/+*^ mice. The resulting *Apc*^*−/+*^*Cdk4*^*−/+*^ were crossed to *Apc*^*+/+*^*Cdk4*^*−/+*^ to generate *Apc*^*−/+*^*Cdk4*^*−/−*^ mice. All animals were maintained on a mixed 129/C57BL/6 background. Pups were genotyped by PCR. For the *Apc* gene, the primers used were A, 5- GACTGCAGATCTCTCCAAG −3; B, 5-CTAAAGCGCATGCTCCAGACT-3; and C, 5-AAGAAGAGCTGGGCAATACCGTA-3. For *Cdk4*^*−/−*^ genotyping, the following primers were used: A, 5- ATATTGCTGAAGAGCTTGGCGG-3; B, 5-CGGAAGGCAGAGATTCGCTTAT-3; and C, 5-CCAGCCTGAAGCTAAGAGTAGCTGT-3. Mice were fed AIN-76A diet and water ad libitum, were exposed to 12-hour-light/12-hour-dark cycles, and were maintained under specific pathogen free conditions including pinworms, *Helicobacter spp*, and *Citrobacter rodentium*. The wild-type *Apc* allele generated a 1171-bp product, and the heterozygous allele formed one wild type allele and one knockout allele, below 1000-bp in size. The wild-type *Cdk4* allele generated a 195-bp product, and the mutant allele formed a 315-bp product. We used age- and sex- matched littermate controls. All experiments on animals were approved in accordance with the Institutional Animal Care and Use Committee at The Johns Hopkins University.

### Analysis of intestinal adenoma

At the age of four months, *Apc*^*−/+*^*Cdk4*^***−/−***^ and *Apc*^*−/+*^*Cdk4*^*+/+*^ female mice were euthanized by cervical dislocation. The intestines were excised, opened, laid flat, washed in phosphate buffered saline, and stained with methylene blue then de-stained with 20% alcohol five to six times to desired color and contrast. The numbers of adenomas were counted using a dissecting microscope in both small and large intestine, blinded as to group. Questionable lesions were examined histologically to confirm them as adenomas. Also, the number of adenomas was classified according to their size (< 1 mm, 1–2 mm, 2–3 mm, > 3 mm). The intestines were rolled, embedded, and sectioned for microscopic examination.

### Immunohistochemistry

The adult *Apc*^*−/+*^ mice were euthanized by cervical dislocation. The intestines were opened longitudinally, washed in PBS, and fixed in 10% buffered formalin. Then the intestines were rolled and submitted for embedding. Five-μm-thick sections were prepared and sections were deparaffinized in xylene, and rehydrated through graded alcohols. Slides were transferred to a jar containing unmasking solution (Vector Laboratories, H-3300), boiled for 10 minutes, and left in the same solution at room temperature for 20 minutes. Endogenous peroxidase was quenched with hydrogen peroxide for 10 min. All slides were then incubated with 10% blocking serum (Vector Laboratories), from the animal species from which the secondary antibody was made, in PBS for 30 min at room temperature to saturate nonspecific protein binding sites. The slides were incubated with primary antibody (Cdk4, pRb, and cyclin D1) diluted 1:100 for 60 minutes at room temperature (Cell Signaling Technology, Cdk4: 2906, pRb: 9308, cyclin D1: 2926). After three washes with 0.1% Tween 20 in PBS, sections were incubated for 30 minutes with biotinylated secondary antibody IgG (Vector laboratories) diluted 1:500 in blocking solution. After three washes, sections were incubated for 30 min with ABC reagent. The slides were then washed for 5 minutes three times. The final reaction was produced by immersing the sections in a peroxidase substrate with nickel enhancer (Vector Laboratories, SK-4100) at room temperature. Controls for immunostaining included incubations with matched rabbit immunoglobulins. Photographs of histological sections were taken using a Nikon digital camera (DXTM1200).

For both proliferation and apoptosis, silibinin and vehicle treated *Apc*^*−/+*^ mice were euthanized at the age of four months and immunohistochemistry was performed as described above. For proliferation assays we used Ki67 primary antibody (Lab Vision Corp, RM-9106-S). For apoptosis: after rehydration, slides were transferred to coplin jars containing Proteinase K (Dako, S3020) at 37°C for 15 min. After washing, TUNEL reagent (*in situ* cell death detection kit fluorescein; Roche #1684795) was added and slides were placed in a humid chamber for 60 minutes at 37°C in the dark. Slides were washed three times in PBS for 3 minutes each time, and rinsed three times with distilled water. Cover slips were mounted with crystal mount (Biomeda, M02). The number of TUNEL and Ki67 positive cells were counted per high power field (400×) per 4 crypts in normal regions of the mucosa and within the adenomas. The quantification was scored, blinded to intervention (treatment/control).

### Growth inhibition assay

HT-29 (2 × 10 ^4^) cells were seeded into each well of a 96-well plate in 100 μL tissue culture DMEM medium supplemented with 10% heat-inactivated fetal bovine serum (FBS), and 2 mmol/L glutamine, at 37°C in a humidified atmosphere containing 5% carbon dioxide and 95% air. After 24-hour incubation to allow cells to adhere, cells were treated with silibinin (Sigma Aldrich), esculetin (Sigma Aldrich), curcumin (Sigma Aldrich), or vitamin D analog (unpublished new analogue) at different concentration (0.1, 1, 10, 100 μM). Cultures were incubated for an additional 24 hours. Inhibition of proliferation was determined by a reproducible colorimetric assay. The key component is (3-{4,5-dimethylthiazol-2-yl}-2,5-diphenyl tetrazolium bromide) or MTT using medium or balanced salt solutions without phenol red (Sigma, M-5655). This assay measures the bioreduction by intracellular dehydrogenases of the tetrazolium compound MTT. MTT was added to the culture wells, and the mixture was incubated for 3 hours at 37°C. Then, MTT was removed and MTT solubilization solution (Sigma, M-8910) was added to the well. Absorbance was measured at 570 nm using a microplate reader. Viability was calculated from relative dye intensity of the mean for duplicate samples and presented as percentages relative to vehicle samples.

In addition, after growing and treating cells with different concentrations of silibinin for 24 hours as described above, the cells were trypsinzed and neutralized by medium, stained with 0.4% trypan blue solution to determine viability by dye exclusion and quantitated using a hemocytometer. All assays were performed with four replicates.

### Detection of inhibition of proliferation by flow cytometry

To quantify silibinin inhibition of proliferation of HT-29 cells, Ki67 staining was used followed by flow cytometry. HT-29 cells were plated in 25 mm dishes. At 50% confluency, cells were treated with different concentrations of silibinin (0, 0.1, 1, 10, and 100 μM). 1 × 10^6^ cells were collected, fixed and permeabilized using the protocol described for the BD Cytofix/Cytoperm Kit (Becton Dickinson). Briefly, cells were resuspended in 100 μl of Cytofix/Cytoperm solution for 15 min at 4°C. Cells were washed twice in 1 × Perm/Wash Buffer, and resuspended in 100 μl of Perm/Wash Buffer. Then, 10 μl Ki67 FITC-conjugated antibody (BD) was added to fixed and permeabilized cells for 30 minutes in the dark at room temperature. Cells were washed and resuspended in staining buffer (BD). Proliferation was analyzed by flow cytometry (Facscalibur, Becton Dickinson). Experiments were performed at least three times.

### Western blotting

HT-29 cells were treated with different concentrations of silibinin, (0.1, 0.5, 1, 10, and 100 μM) or with vehicle (DMSO) for 24 hours. To prepare whole cell extracts for *in vitro* study, HT-29 cells treated with silibinin were washed with TBS and suspended in lysis buffer containing protease/phosphatase-inhibitor cocktail (Cell Signaling Technology, 5872). After mixing for 30 minutes at 4°C, the mixture was centrifuged for 10 minutes, and supernatants were collected. For *in vivo* studies 0.2% of silibinin and control diet were given to *Apc*^*−/+*^ mice for five days. The purification of the mucosal lining of the intestine was performed as described in reference [22] with mild m odification. Briefly, after euthanizing the animal, the small intestines were excised and a 5 cm bowel segment from duodenum, jejunum, and ileum were opened on ice and washed with ice cold TBS. All three pieces were placed in a tube containing 0.14 M NaCl containing 5 mM EDTA (pH 7.4). The tubes were vortexed for 30 minutes at 4°C. Detached mucosal cells from all pieces were collected by centrifugation at 800 g for 30 seconds at 4°C and suspended in 10 ml of buffer containing 12 mM Tris-HCl (pH7.4), 0.3 M mannitol, 10 mM KCl, 0.5 mM EDTA, and a protease/phosphatase-inhibitor cocktail. The protein content from the intestinal mucosal cells and the supernatants from HT-29 cell line were determined using the Biorad protein assay reagent with bovine serum albumin as a standard. Thirteen μg of protein were separated on 6–10% Novex Bis-Tris Gel (Invitrogen) and transferred onto a nitrocellulose membrane. Loading of equal protein amounts was assessed by the Biorad assay. The rest of the procedure followed according to the manufacturers protocol (Cell Signaling Technology). Briefly nonspecific binding to the membrane was blocked for 1 hour of incubation with agitation at room temperature. Anti-pRb, anti-Rb, anti-cyclin D1, anti-Cdk4 (1:1000), and anti-actin (1:400) antibodies were added overnight at 4°C. All antibodies were purchased from Cell Singling Technology, 9308, 9309, 2926, 2906, and 8456 respectively. The horseradish peroxidase-conjugated goat anti-rabbit secondary antibody was incubated at 1:2000 for 1 hour at room temperature. Following three washes with Tris-buffered saline, the protein bands were visualized with LuminGLO for 1 min at room temperature. The bands were visualized on film (Kodak) exposed to the membrane to detect chemiluminescence signals. Experiments were repeated three times.

### Treatment protocol

At the age of 30 days, the *Apc*^*−/+*^ mice (groups of 11) were fed either with 0.2% silibinin (w/w) in the diet (AIN-93G, gamma irradiated ground meal diet) or placebo (diet alone) for three months. The diet was made fresh every week and stored at −20°C in the dark to minimize photo destruction of nutrients and silibinin. Fresh diet was provided every three days at the time of cage change. The weight of the mice was recorded twice a week. At the age of 4 months, the mice were euthanized; serum chemistry and hematology were evaluated by a commercial laboratory (Antech Inc.). Adenomas were counted as described previously at the age of four months.

### Data analysis

Data are presented as the mean and standard error of mean or as a percentage with standard deviation (GraphPad, PRISM software, San Diego). The Mann-Whitney Test was used to compare body weights, and *P* values were determined by using the Student two-tailed *t* test unless otherwise indicated. Tumor data were analyzed using Chi square (*χ*^2^) test and other differences using the Student’s *t* test. Differences were considered statistically significant at p < 0.05.

## Results

**Figure 1:**
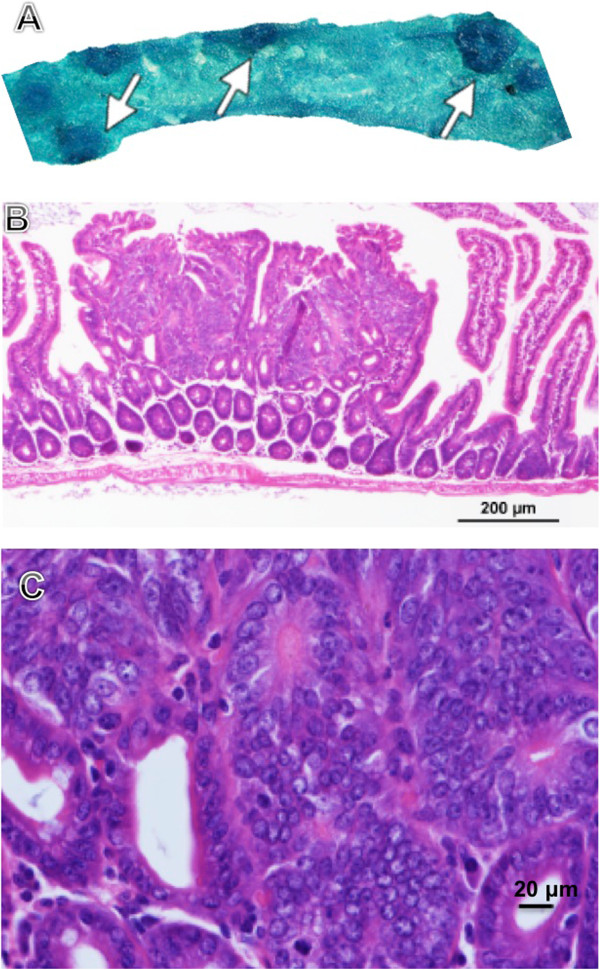
**Macroscopic dissection and microscopic views of the adenomas. A**: Numerous adenomas (arrow) of various sizes observed within the intestine of an *Apc*^*−/+*^ mouse. **B**: Histomorphological features of the small intestinal adenoma, H&E staining. **C**: Higher power view of the adenoma.

**Figure 2:**
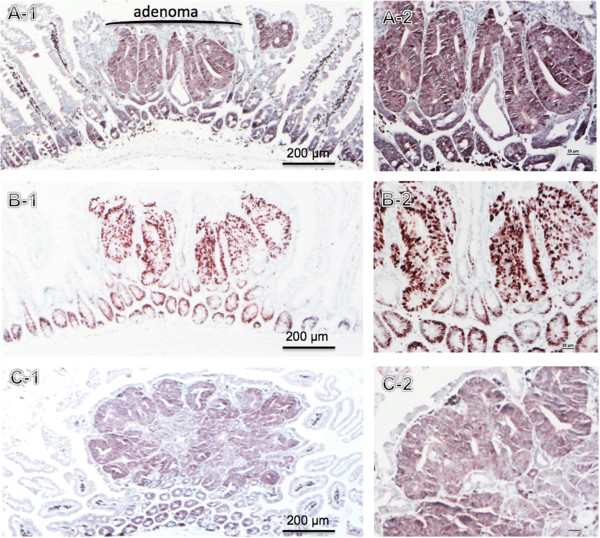
**Immunohistochemical staining of Cdk4 (A), cyclin D1 (B), and pRb (C) in intestine.** Left: Low power view (50×) showing normal intestinal mucosa and adenoma. Right: higher magnification (200×) of the intestinal adenoma.

Figure 1 shows hematoxylin and eosin staining of the intestine and adenomas from mutant *Apc*^*−/+*^ mice. Histologically, adenomas were characterized by proliferation of variably dysplastic epithelium, with minor differentiation into mature goblet or absorptive cells [23]. Intestines from *Apc*^*−/+*^ mice were analyzed by immunohistochemistry for the expression and distribution of three central components of the Cdk4 signaling pathway downstream of *Apc* mutation, Cdk4, cyclin D1, and pRb. The results show that all mice had nuclear staining for all three markers within the nuclei from normal crypts as well as from adenomas (Figure 2A, B, and C). There were differences in the intensity of the staining in different mice in some instances for the same marker. The pattern of Cdk4 and cyclin D1 staining was strongest in the crypts and adenomas and almost absent from the villi. For pRb, there was stronger staining in the crypts and adenomas with weak staining of the villi. We conclude that indeed there is prominent expression of Cdk4 pathway components throughout adenoma epithelium.

**Figure 3:**
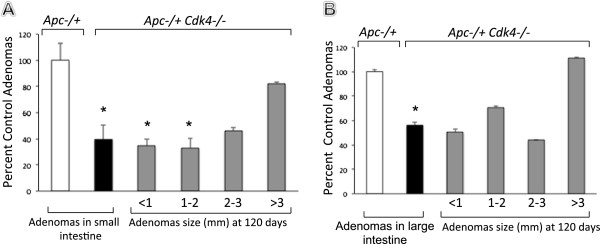
**Distribution of adenomas by size in *Apc***^***−/+***^***Cdk4***^***+/+***^**(white bar) and in *Apc***^***−/+***^***Cdk4***^***−/−***^**mice (filled bar) in small (A) and large (B) intestine.** The percent of control adenomas are indicated with SEM. The number of adenomas were analyzed by the Chi square test and Student *t* test. (*p < 0.05 versus control. *n* = 15 animals/group).

To further define a potential role for Cdk4 blockade in preventing intestinal tumor formation, we generated double mutant mice, *Apc*^*−/+*^*Cdk4*^*−/−*^*,* and compared them to control *Apc*^*−/+*^*Cdk4*^*+/+*^ mice. Adenomas were counted manually as described in the materials and methods section using a dissecting microscope. Double mutant mice exhibited significantly reduced adenomas, compared with *Apc*^*−/+*^*Cdk4*^*+/+*^ mice along their entire small and large intestine at 120 days of age. The lack of Cdk4, reduced the sizes of all adenomas by 65%. The number of microadenomas that were less than 1 mm in diameter were significantly decreased in double mutant mice, *Apc*^*−/+*^*Cdk4*^*−/−*^*,* (Figure 3).

We next compared several potential candidate compounds for their effectiveness as inhibitors of Cdk4 signaling and cell proliferation *in vitro*. We found that among esculetin, curcumin, a vitamin D analogue, and silibinin, all inhibited the Cdk4 pathway at various doses. However, silibinin possessed the best combination of efficacy in inhibiting Cdk4 signaling and lack of toxicity in treated cells at the effective dose. Esculetin, curcumin, and vitamin D analogue treated cells exhibited detectable toxicity (reduced viability) at doses that displayed evidence of Cdk4 pathway inhibition and therefore were not studied further.

**Figure 4:**
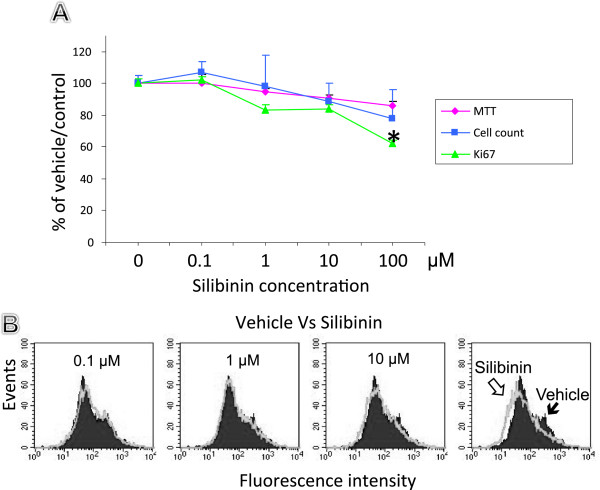
**Cell growth, viability, and Ki67 staining of the silibinin treated HT-29 cells. A**: HT-29 cells were incubated with vehicle (DMSO) and silibinin, at 0, 0.1, 1, 10, and100 μM and cells or events counted at 24 hours. Results shown are the average of triplicate determinations. Results are expressed as the mean and normalized against their own control, vehicle. (Bar = SEM. * *P* < 0.05 versus control). **B**: Analysis of proliferation in HT-29 treated cells by flow cytometry.

To further define how silibinin acts, cell number and viability was monitored over time in culture as a function of mitochondrial activity in living cells using the MTT assay. The inhibition of proliferation for silibinin treated cells was clearly dose dependent (Figure 4). A reduction of proliferation was statistically significant at 100 μM in cells treated with silibinin. A similar pattern was noted when we counted viable HT-29 cells using the trypan blue assay and a hemocytometer. In addition, the Ki67 labeling index confirmed the effects of silibinin on cell growth as measured by flow cytometry. The inhibition of expression of Ki67 in HT-29 cells was strongest at 100 μM. While the expression of Ki67 in HT-29 treated cells at 0.1 and 1 μM was almost similar to non-treated cells.

**Figure 5:**
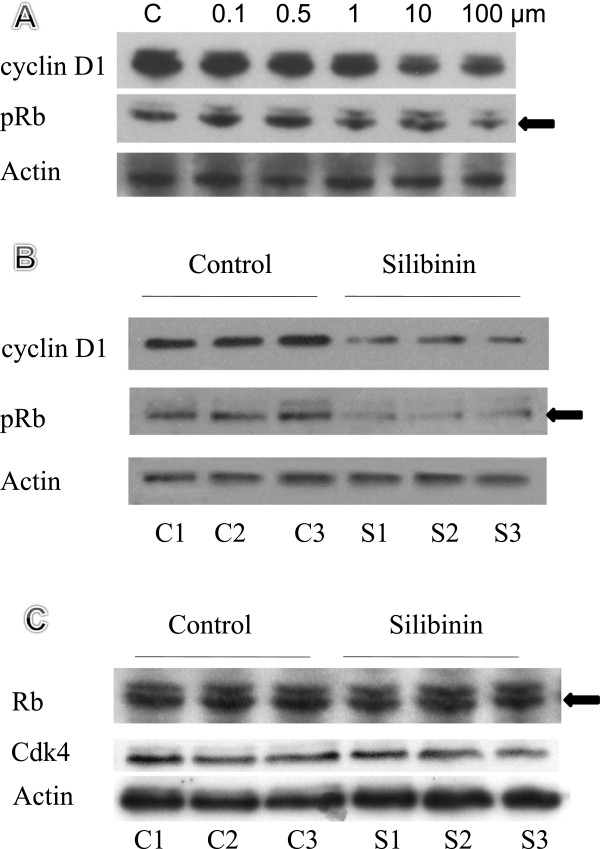
**Western blot analysis of pRb, cyclin D1, Cdk4, and Rb. A**: Analysis of cyclin D1 and pRb in total cell lysates from HT-29 cell line treated with 0.1, 0.5, 1, 10, 100 μM of silibinin. **B** and **C**: Analysis of cyclin D1, pRb, Cdk4, and Rb of the mucosal cells lining the intestine after silibinin administration for 5 days. The blots were re-probed using β-actin as a loading control.

To further evaluate Cdk4 pathway activity, we measured downstream phosphorylated Rb (pRb) and binding partner cyclin D1 in whole cell lysates isolated from viable HT-29 cells treated with different concentrations (0.1, 0.5, 1, 10, and 100 μM) of silibinin. Proteins were examined by Western blot using chemiluminescence for detection. Results in Figure 5A show there is significant, dose-dependent reduction in levels of phosphorylated Rb and cyclin D1. This indicated that silibinin causes hypophosphorylation of Rb protein and also reduces cellular levels of cyclin D1.

Next, an *in vivo* study was performed to see whether silibinin had a similar effect on phosphorylation of Rb in intestinal mucosal tissue. Western blot analysis was performed on the protein extracted from the mucosa from mice that were fed control diet or a diet containing 0.2% silibinin in the food. The phospho-specific pRb and cyclin D1 bands were reduced significantly in animals receiving silibinin (0.2%) for five days (Figure 5B). Rb and Cdk4 protein levels were the same in silibinin-treated and non-treated mice (Figure 5C).

**Figure 6:**
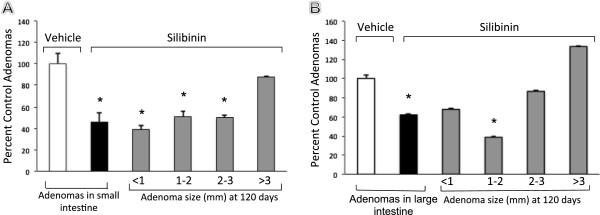
**Distribution of adenomas by size in *Apc***^***−/+***^**(white bar) and in *Apc***^***−/+***^**mice that received silibinin (filled bar) in small (A) and large (B) intestine.** The percent of control adenomas are indicated with SEM. The number of adenomas were analyzed by Chi square test and Student *t* test. (*p < 0.05 compared with control. *n* = 11 animals/group).

To determine if silibinin was effective in preventing adenomas, a known precursor to colorectal cancer, we next performed a chemopreventive study using the *Apc* mouse model. Our data show that a diet containing 0.2% silibinin significantly reduced the number of large and small intestinal adenomas (Figure 6). The total number of adenomas decreased by 52% in silibinin treated mice compared to those with vehicle only. The number of adenomas that were less than 2 mm in diameter were significantly decreased in silibinin treated animals. The adenomas that were larger than 2 mm in diameter were also reduced in silibinin treated animals; however, the reduction did not reach significance. The body weight gains, gross examination, and microscopic examination, serum chemistry, and blood counts were within normal limits with no indications of toxic side effects at the dosage of silibinin that was used (data not shown).

**Figure 7:**
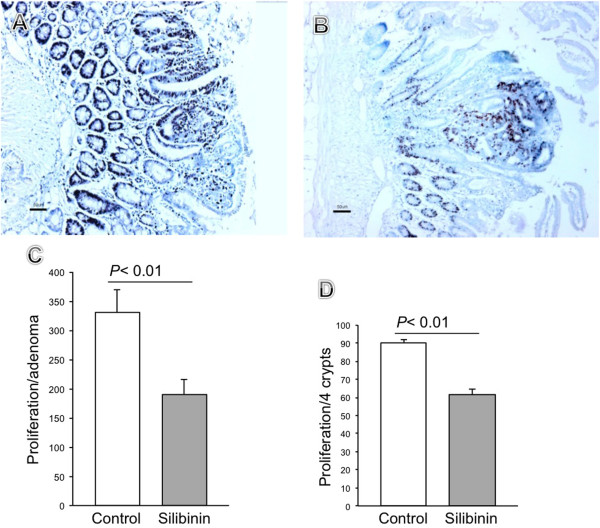
**Silibinin affects Ki67 expression in *Apc***^***−/+***^**mice.** Representative examples of immunohistochemical analysis of Ki67 in *Apc*^*−/+*^ control (**A**) and silibinin treated mice (**B**). Quantification of Ki67 immunostaining within the adenomas (**C**) and within the crypts (**D**) in control (empty bar) and silibinin treated mice area (filled bar). (*p < 0.01 compared with control, by *t* test. n = 4 animals/group).

Cell cycle activity in the intestinal epithelium in silibinin treated and control *Apc*^*−/+*^ mice were compared using immunohistochemistry for Ki67 (Figure 7). Normal crypt Ki67 staining was substantially reduced (33%) in mice that were on silibinin comparing to the mice that received control diet. The mean proliferation index within the normal crypts in the mice that were on silibinin was 61 but was 90 in the mice that received no intervention, (p < 0.01 in *t* test). Also, there was strong repression of Ki67 positive cells within the adenomas from the mice that were on silibinin. In addition, the mean proliferative index within the adenomas was 191 in the mice that were on silibinin but 330 in the mice received control food, (P < 0.01 in *t* test) (Figure 7).

**Figure 8:**
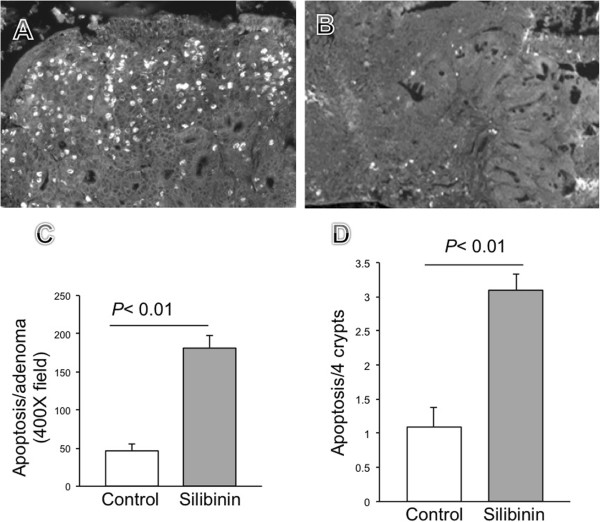
**Immunofluorescent detection of apoptotic cells in intestinal adenoma.** Representative analysis of tunnel staining in *Apc*^*−/+*^ control (**A**) and silibinin treated mice (**B**). Quantification of tunnel positive cells within the polyps (**C**) and within the crypts (**D**) in control (empty bar) and silibinin treated mice area (filled bar). (*p < 0.01 compared with control, by *t* test. *n* = 4 animals/group).

Apoptosis of intestinal epithelial cells was characterized in intestinal tissue histologically according to an apoptotic index using the TUNEL assay. Apoptosis was found in the crypts, epithelial cells on the small intestinal villi, crypts, and intercrypt table of the colon. The results demonstrated that the mice that were on silibinin had significantly increased numbers of apoptotic cells (average per field 3.1) in crypts in the normal mucosa compared to the mucosa in mice that were on control diet only (average 1.1 per field) (Figure 8). Similarly the number of apoptotic cells was higher within adenomas in the mice that were on silibinin (180) compared to the mice that received no intervention (48) (Figure 8). These differences between silibinin treated and control mice that were seen in both the crypts in the unaffected mucosa and in the adenomas were statistically significant, (p value < 0.01).

## Discussion

Mutation in the *Apc* gene leads to nuclear accumulation of β-catenin and over expression of cyclin D1 and increased Cdk4 activity [8,10]. We have shown that cyclin D1, and Cdk4 expression is markedly reduced in mature epithelium at the luminal surface but clearly present within adenomas. Modulation of these proteins may provide effective chemoprevention against colorectal cancer. Normally the intestine is covered by a mucosal layer that is composed of crypts deep in the mucosa and villi (small intestine) or the intercrypt table (large intestine) at the luminal surface [24]. Epithelial stem cells are located near the base of the crypts [25,26]. Epithelial cells undergo proliferation and differentiation as they migrate up the crypt wall towards the lumen [27]. Mutations that occur in stem cells allow for persistence and accumulation of the mutations through stem cell self-renewal. Therefore, mutations in the stem cells pass to the future generations of stem cells and progeny daughter cells.

*Apc* mutant mice develop large numbers of adenomas in their small intestine and fewer in the large intestine whereas; FAP patients develop low numbers of adenomas in their small intestine and large numbers of adenomas in their large intestine. In spite of differences in localization of adenomas, it has been widely used as a predictive model for human colorectal cancer [28-30].

In our study, we first used a genetic approach to explore the role of *Cdk4* in reducing intestinal tumors in *Apc*^*−/+*^ mice. The results of this study revealed that the disruption of Cdk4 activity in *Apc*^*−/+*^ mice led to a significantly reduced number and size of tumors. We found that *Apc*^*−/+*^*Cdk4*^*−/−*^ mice had fewer tumors of all sizes that are less than 3 mm in diameter compared to *Apc*^*−/+*^*Cdk4*^*+/+*^ mice. This demonstrates that the *Cdk4* pathway is important in the development or maintenance of adenomas in *Apc*^*−/+*^ mice. Since the smallest microadenomas were reduced in number, *Cdk4* may affect initiation or very early events of microadenoma formation in mice heterozygous for *Apc*. Thus, earliest microadenomas were reduced in number due to reduced Cdk4 activity. The loss of *Cdk4* leads to hypophosphorylation of Rb and decreased tumorigenesis. Several studies have demonstrated the contribution of *Cdk4* in tumor development in brain, skin, pancreas, eye, and tooth [31-33]. We conclude that *Cdk4* is a potential target for blockade in the chemoprevention of colorectal cancer.

In our *in vitro* studies, the effects of silibinin on growth inhibition of intestinal tumors was demonstrated. We also observed an associated effect of silibinin on Rb phosphorylation and cyclin D1 levels in HT-29 cell lines and *in vivo* in intestinal tissue. It is possible that silibinin directly reduces Cdk4 activity in addition to loss of it’s binding partner cyclin D1 that leads to hypophosphorylation of Rb. These results suggest that silibinin may cause cell cycle arrest at the G_0_ to G_1_ stages of the cell cycle due in part to its targeting of the Cdk4 signaling pathway (Cdk4, cyclin D1, pRb) at multiple steps. The silibinin effects included significant alterations in cell cycle regulator proteins causing cell cycle arrest followed by cell growth inhibition and apoptotic cell death. Other studies have shown that G1 arrest was related to strong induction of Cip1/p21 and Kip1/p27 protein levels and a significant decrease in Cdk2, Cdk4 and their associated cyclin E and cyclin D1 protein levels following HT-29 treatment with silibinin [34]. *Apc*^*−/+*^ mice are a widely accepted preclinical model to evaluate the antitumor efficacy of candidate chemopreventive agents for colorectal cancer and to determine associated toxicity [35]. In this study examination of the very early stages of colorectal neoplasia (formation of small microadenomas) was assessed *in vivo*. Dietary supplementation with silibinin (0.2%) resulted in a decrease in activity of the Cdk4 pathway and markedly reduced adenoma formation in *Apc*^*−/+*^ mice. The smallest tumors (< 1 mm) were decreased in number suggesting that silibinin may have inhibited microadenomas at or shortly after they were initiated as well as inhibiting adenoma growth. Other studies have show that in the colon, dietary feeding of silibinin reduced the occurrence of aberrant crypt foci in azoxymethane (AOM) treated rats and induced GST and NQO1 [36]. Sangeetha et al. and Gershbein et al. found that dietary feeding of silymarin significantly inhibited development of intestinal adenocarcinomas in DMH treated rats [37,38]. In addition, Rajamanickam at al. showed that silibinin significantly inhibited adenoma formation in *Apc* mutant mice by inhibiting cell proliferation [39]. However, no studies have explored mechanistically the antiproliferative activity of silibinin on the Cdk4 pathway in *Apc* mutant mice.

Our *in vivo* results show that silibinin can significantly inhibit cell proliferation in the crypts and adenomas in the intestine in *Apc*^*−/+*^ mice as evidenced by Ki67 staining. Cell proliferation depends on a tightly regulated system involving multiple genes, many of which have been implicated in multistep carcinogenesis. This is consistent with many studies that have suggested that silibinin induces growth inhibition and promotes cell cycle arrest in many CRC cell lines [17,34,40,41]. Increased TUNEL-positive cells were noted within the crypts in the normal, nonpolypoid mucosa in mice that received silibinin as well as in adenomas. It was previously shown that silibinin causes apoptotic cell death in SW480 and SW620 cells [41,42]. Many studies have shown that elimination of transformed cells via apoptosis in the colon is an important step to restore normal epithelial growth [42,43]. Alterations in apoptosis also have profound effects on the progression of benign to malignant tumors [44,45]. In summary, disruption of the homeostatic balance between proliferation and apoptosis has been broadly implicated in cancer and silibinin tips the balance away from development of colorectal cancer.

**Figure 9:**
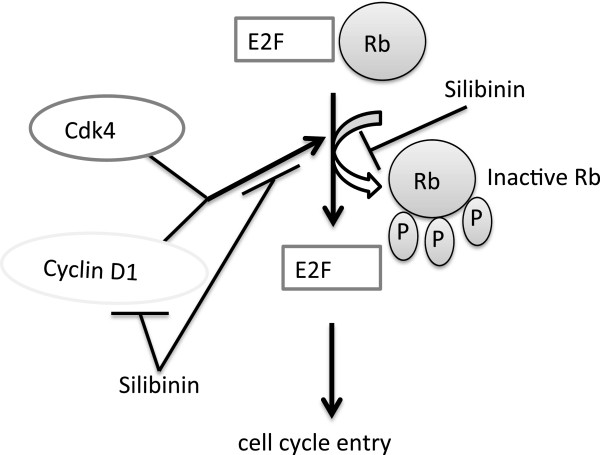
**Schematic model of potential silibinin effects on the Cdk4 pathway in controlling tumor formation.** Blockade of Cdk4 pathway (cyclin D1, Cdk4 activity, and pRb) and inhibition of cell cycle entry.

Rajamanickam et al. have suggested that silibinin has multiple mechanisms potentially driving its anticancer effects in *Apc*^*−/+*^ mice. These effects include apoptosis, decreasing beta-catenin levels and transcriptional activity, and modulating the expression profile of cytokines [39]. Also, silibinin inhibits PP2A/AKT/mTOR pathways and targets the inflammatory NF-kB pathways suppresses colorectal cancer stem like cells [46,47]. In the present study, silibinin has inhibitory effects on the Cdk4 pathway and appears to mediate at least some of its effects through the cell cycle, proliferation of adenomas, and apoptosis (Figure 9). The relative importance of each Cdk4 pathway target for silibinin activity in altering intestinal epithelial cells remains to be fully elucidated.

## Conclusions

In conclusion, results described in this report suggest that the inhibition of the Cdk4 pathway as evidenced by hypophosphorylation of Rb protein contributes to the antitumor activity of silibinin and the chemopreventive ability of silibinin is likely related in part to its suppression of cell proliferation or augmentation of apoptosis of mutated intestinal epithelial cells. Therefore, silibinin may serve as a non-toxic chemopreventive agent for colon cancer and it clearly deserves further consideration for colorectal cancer prevention studies.

## Competing interests

The authors declare that they have no competing interests.

## Authors’ contributions

BOK: designed and performed all experiments and wrote the manuscript. KJR, GL, and DZ assisted in experiments. DLH edited the manuscript and conceived the project. BOK, KJR, and DLH contributed to the analysis of data. All authors read and approved the final manuscript.

## Pre-publication history

The pre-publication history for this paper can be accessed here:

http://www.biomedcentral.com/1471-2407/13/157/prepub
